# Magnetic particles in motion: magneto-motive imaging and sensing

**DOI:** 10.7150/thno.54056

**Published:** 2022-01-24

**Authors:** Kelsey P. Kubelick, Mohammad Mehrmohammadi

**Affiliations:** 1 Wallace H. Coulter Department of Biomedical Engineering, Georgia Institute of Technology and Emory University School of Medicine, Georgia, USA; 2 School of Electrical and Computer Engineering, Georgia Institute of Technology, Georgia, USA; 3 Department of Biomedical Engineering, Wayne State University, Michigan, USA; 4 Barbara Ann Karmanos Cancer Institute, Michigan, USA

**Keywords:** Magnetic nanoparticles, magneto-motive contrast, biomedical imaging, diagnostics, elastography

## Abstract

Superparamagnetic nanoparticles have become an important tool in biomedicine. Their biocompatibility, controllable small size, and magnetic properties allow manipulation with an external magnetic field for a variety of diagnostic and therapeutic applications. Recently, the magnetically-induced motion of superparamagnetic nanoparticles has been investigated as a new source of imaging contrast. In magneto-motive imaging, an external, time-varying magnetic field is applied to move a magnetically labeled subject, such as labeled cells or tissue. Several major imaging modalities such as ultrasound, photoacoustic imaging, optical coherence tomography, and laser speckle tracking can utilize magneto-motive contrast to monitor biological events at smaller scales with enhanced contrast and sensitivity. In this review article, an overview of magneto-motive imaging techniques is presented, including synthesis of superparamagnetic nanoparticles, fundamental principles of magneto-motive force and its utility to excite labeled tissue within a viscoelastic medium, current capabilities of magneto-motive imaging modalities, and a discussion of the challenges and future outlook in the magneto-motive imaging domain.

## Introduction

One of the first accounts of a naturally magnetized material was a lodestone, documented by Greek philosophers in 6^th^ century B.C. The unexplained magnetic force was originally associated with magic and the occult, but was later used in early magnetic compasses 1. Today, many types of magnetic materials have been synthesized and engineered for a variety of applications, especially in the field of nanotechnology. Superparamagnetic properties were discovered upon reducing the size of magnetic materials to the nanoscale 2. Superparamagnetism, in addition to size and shape effects, has generated many opportunities for magnetic nanoparticles (MNPs) in biomedicine 3-7. MNPs are set to become an integral part of diagnostics, gene and drug delivery 8, cellular therapies 9-11, hyperthermia 12-18, and imaging 19-21.

**Figure 1:**
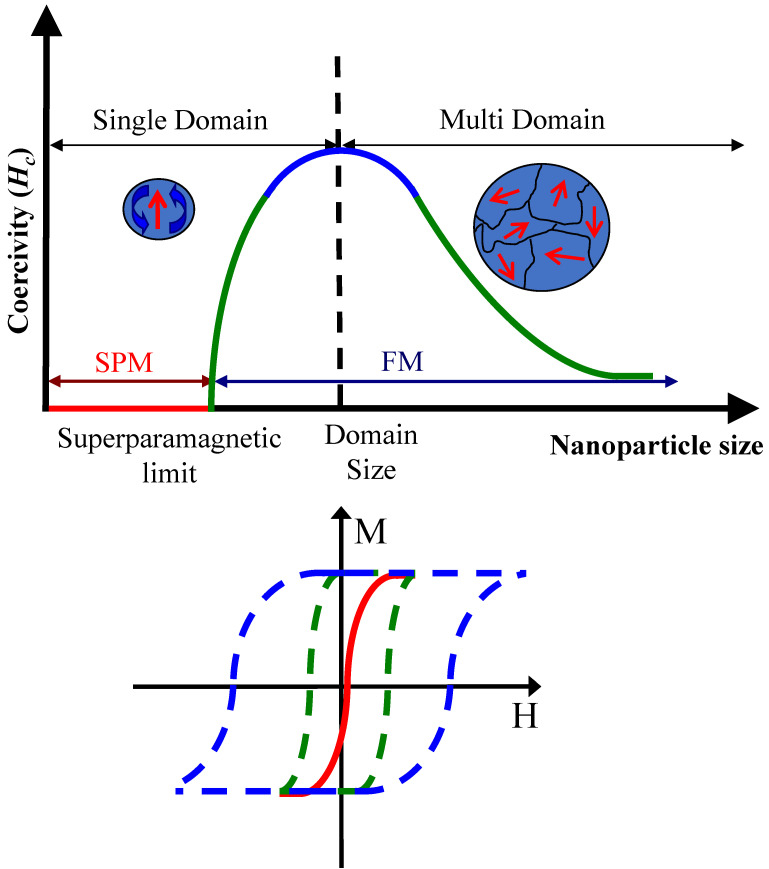
** Illustration of the critical size effect of superparamagnetic nanoparticles**. As particle size is reduced below the critical size, superparamagnetic (SPM, solid red line) nanoparticles are only magnetized in response to an externally applied magnetic field. In comparison, ferromagnetic (FM, dashed lines) nanoparticles are permanently magnetized. H represents the applied magnetic field strength, and M is the measured magnetization. Reproduced with permission from 22. Copyright 2010, IOP Publishing.

Permanently magnetized particles are generally composed of ferromagnetic or ferrimagnetic materials, such as magnetite (Fe_3_O_4_) and maghemite (Ɣ-Fe_2_O_3_). When particle size is reduced, magnetic properties of ferro- and ferrimagnetic materials change. Below the critical size threshold, MNPs magnetize in response to an applied external magnetic field, but have low remnant magnetization upon removal of the magnetic field (Figure 1) 22. This characteristic is similarly observed in paramagnetic materials. Superparamagnetic materials are distinguished by their larger magnetic susceptibility and smaller size 2,7,23-25. Superparamagnetism allows for design of MNPs that are on the same size-scale as biological entities while exhibiting strong magnetic properties. Following removal of the external magnetic field, minimal residual magnetization allows clearance of MNPs and prevents particle agglomeration 6,26. For these reasons superparamagnetic MNPs have generated substantial interest for biomedical research, especially in imaging applications.

Superparamagnetic MNPs have been widely used as contrast agents in various diagnostic applications of magnetic resonance imaging (MRI), including imaging of atherosclerotic plaques, stem cell treatments, cancer, and drug delivery 20,27,28. Magnetic contrast is generally appealing due to the ability to detect biological targets with high sensitivity and contrast while suppressing background signals. Although MRI has well-established use in clinic and has tremendous capabilities for diagnostic imaging, it is not ideal for all applications. The utility of MNPs as cellular and molecular contrast agents can be expanded beyond MRI. Magnetic particle imaging (MPI) is another method that directly leverages magnetic contrast 19,29. An alternative strategy is to develop indirect methods of detecting MNPs. The latter approach can expand the utility of magnetic contrast to many traditionally non-magnetic imaging modalities and is a major motivating factor behind development of magneto-motive imaging techniques 30,31.

In magneto-motive imaging, contrast is generated through magnetically-induced motion 30-35. When an external magnetic field is applied to a target labeled with MNPs, the magnetically-labeled target undergoes distinct motion compared to the background tissue. The background area adjacent to the magnetically-labeled tissue experiences shear motion. The magneto-motive driven motion, which is primarily compressional motion, not shear motion, can be easily distinguished from the background using time gating for pulsed excitation or phase locking methods for harmonic excitation. Following magnetic excitation, removing the external magnetic field returns the magnetically-labeled target to its original equilibrium position due to mechanical resistance of the surrounding viscoelastic tissue. In summary, changing the magnitude (cyclic *B*) or turning the external magnetic field repeatedly on and off (pulsed *B*) generates contrast based on magnetic movement. As a result non-magnetic modalities, such as ultrasound (US), optical coherence tomography (OCT), photoacoustic (PA) imaging, and laser speckle tracking (LST), can be used to measure magnetic motion of MNPs and, therefore, create diagnostic value and new imaging opportunities.

There are two forces that are critical to producing magneto-motive contrast: 1) magneto-motive force and 2) opposing viscoelastic force of the surrounding tissue 32,36,37. Magneto-motive force describes the response of the MNP to the applied external magnetic field. Viscoelastic force describes the opposition of the surrounding tissue to displacement, causing the MNP to return to its equilibrium position upon removing the external magnetic field. Together, magneto-motive and viscoelastic forces create the oscillatory movement required to produce magneto-motive contrast. *Equation 1* describes the magneto-motive force, F_m_ 38,39:


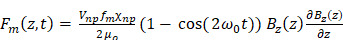

(1)

where V_np_ is the volume of the MNP, f_m_ is the volumetric ratio of magnetic material, 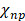 is the magnetic susceptibility of the MNP, and B_z_ is the magnetic flux density. *Equation 2* combines the magneto-motive force and the viscoelastic force of the surrounding tissue to describe the complete magneto-motive system 38,39:


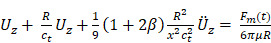

(2)

where *U* is the displacement vector of the particle-labeled tissue, *R* is the radius of the MNP, and 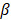 is the normalized nanoparticle density. Shear wave speed is defined as 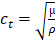, where 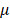 is the shear elasticity coefficient, 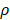 is the density of the surrounding medium, and *t* is time. Equation 2 was simplified to only consider motion in the z-dimension, which can be implemented by controlling the spatial gradient of the external magnetic field 38,39.

There are several important observations when analyzing the relationship between magneto-motive force and magnetically induced displacement. In terms of the magnetic field, a greater applied field and gradient increase magnetically induced motion. In terms of the particle, magneto-motive force linearly increases with magnetic susceptibility, volume of the nanoparticle, and the volumetric ratio of magnetic material. Therefore, customization of particle geometry, shape, and material impacts magneto-motive contrast 25,31,40. Overall the principles of magnetism, magneto-motive force, and viscoelastic force directly inform development of magneto-motive imaging platforms for biomedical applications.

In this review we briefly describe nanoconstruct synthesis and design, followed by an overview of magneto-motive (MM) imaging modalities, including MMUS, MM-OCT, MM-LST, magneto-photoacoustic (MPA) imaging, and MM elastography. Finally, we discuss current challenges and future outlook of the magneto-motive imaging field.

## Synthesis of superparamagnetic nanoparticles (SPIONs)

Magnetic nanoparticles are a critical component of magneto-motive imaging platforms. Particle composition, including size, shape, and material directly impact magnetic properties and therefore magneto-motive contrast. Many other review articles provide highly detailed information on MNPs and synthesis 3,41-44. Here, we focus on MNP composition specifically related to developing magneto-motive imaging platforms for biomedical applications.

Superparamagnetism is a key characteristic of magneto-motive imaging contrast agents (Figure 1). High magnetic susceptibility and lack of remnant magnetization results in substantial movement in response to an external magnetic field, which ultimately results in high magneto-motive contrast. The critical size at which permanently magnetized materials begin to show superparamagnetic qualities varies based on particle size, shape, material, and composition 42. Iron and its oxidized forms, magnetite and maghemite, are highly favored materials for nanoparticle synthesis due to their abundance in nature, high magnetic saturation, and high Curie temperature 3.

Superparamagnetic iron oxide nanoparticles (SPIONs) are one of the most commonly used magneto-motive imaging contrast agents. In terms of magnetic properties, using the largest particle possible while still maintaining superparamagnetic qualities is desirable. Larger nanoparticles have greater magnetization, which corresponds to greater particle movement to improve contrast. However, the impact of SPION size on particle clearance and tissue targeting must also be considered for biomedical applications 26. Many types of SPIONs are currently under clinical investigation for MRI. Similar particle formulations could be applied to magneto-motive imaging platforms 28,45-47. The review from Dadfar et al. provides an excellent summary of formulations of iron oxide nanoparticles that are clinically approved or under clinical investigation for different applications 46. We briefly summarize details of the aforementioned review here. Iron oxide nanoparticles below 30 nm are being investigated for metastatic lymph node imaging, cellular labeling, macrophage imaging, neural imaging, cardiovascular imaging, hepatic imaging, and as blood pool agents. Iron oxide nanoparticles between 60 nm - 180 nm are being investigated for liver imaging. Iron oxide nanoparticles between 300 nm - 3500 nm diameter are being investigated for gastrointestinal imaging following oral administration. In the above examples, size is often reported as a hydrodynamic diameter, which takes into account particle coating. Various particle coatings can be used to increase biocompatibility, alter cell-particle interactions, stabilize nanoparticles, allow functionalization, or alter magnetic properties 44,46,48,49.

To further enhance magnetic properties, particle shape, geometry, and size can be modified. The impact of particle shape on magnetic properties has not been extensively studied, but some research shows changes in shape lead to shape anisotropy, which impacts coercivity, magnetic susceptibility, and directionality of the external magnetic field 42,43,48. For example, cobalt nanowires and nanorods had different remnant magnetization and coercivity depending on particle orientation in the magnetic field 50. In another study, MNPs with a cubic shape had higher relaxivity compared to spherical counterparts 51. Particle shape also impacts reactivity, stability, and size - shaped nanoparticles tend to be larger. Though substantial investigation remains to fully understand the impact of shaped MNPs on magnetic properties, various types have been synthesized, including nanocubes 51,52, nanorods 53, nanowires 50, nanodiscs 54,55, tetrapods 56,57, core-shell nanoparticles 58,59, nanoworms 60, and nanoflowers 61,62.

Beyond iron oxides, MNPs can be doped with d-block elements to create a large magnetic moment, including cobalt (Co), manganese (Mn), or zinc (Zn), or rare earth metals, like gadolinium (Gd) or holmium (Ho) 43,63. Development of composite particles is also gaining momentum. One defining characteristic of composites is the ability to generate multimodal contrast. Several nanocomposites have already been developed for MPA imaging, which requires optical and magnetic contrast. Examples include iron/gold core-shell nanoparticles 64,65, liposomes loaded with gold nanoparticles and iron oxide nanoparticles 34,36, gold nanoparticles decorated with SPIONs 66, and Prussian blue nanocubes 67-69.

Safety must always be considered when developing nanoparticle contrast agents due to potential genotoxicity, induction of apoptosis, organ toxicity, blood compatibility, immune response, and impact on cell proliferation 70. Cytotoxicity is often caused by generation of reactive oxygen species through direct reactions at the nanoparticle surface, leaching of iron molecules from the nanoparticle, altering organelle function, or activating signaling pathways with cytotoxic effects. Thus, an inert, non-reactive nanoparticle surface is important 19,71. Surface coatings, such as dextran, can help address these issues 70. Regarding clearance, particles below 10 nm can be removed through extravasation and renal clearance, whereas larger particles are localized to the spleen. Upon cellular uptake, iron oxide nanoparticles are often localized to endosomes and are degraded into free iron, which may pose additional concerns 72,73. However, safety issues can be addressed through proper design controls 46. Some formulations of MNPs are clinically approved, and FDA-approved agents have minimal risk of causing adverse events at appropriate doses 74.

As magneto-motive imaging techniques advance towards the clinic, translation may be expedited by integrating magneto-motive contrast with existing imaging technologies. Therefore, composite nanoparticles that were developed to combine magnetic and non-magnetic modalities can extend to magneto-motive imaging. One example is microbubbles tagged with iron oxide nanoparticles for ultrasound and MRI 75. Iron-laden microbubbles also add therapeutic benefits for applications like blood-brain-barrier opening or drug delivery 76. In another example, ^68^Ga-labelled magnetic nanoparticles combine positron emission tomography (PET)/computed tomography (CT), MRI, and MMUS 77. Although SPIONs have been the particle-of-choice for magneto-motive imaging thus far, there is great opportunity to tailor nanoconstruct design by changing particle size, shape, surface coating, and material. Combining these qualities in clever ways can create contrast agents that are well-suited for different biomedical applications of magneto-motive imaging and theranostics.

## Magneto-motive-based imaging modalities

There are several different magneto-motive imaging modalities. Although magnetic-induced motion is central to all, each has distinct advantages and is preferable for different applications. Magneto-motive ultrasound 39,40,78-80, MM-OCT 32,81-89, MPA imaging 34,36,66,90-93, and MM-LST are reviewed below 94,95, including an overview of the traditional non-magnetic imaging modality, how the modality is modified to generate magneto-motive contrast, example applications, and current challenges. Beyond imaging, magneto-motive contrast can be further extended to functional monitoring and diagnostics, for example, through tissue elasticity measurements 88,96-103.

### Magneto-motive / pulsed magneto-motive ultrasound (MMUS/pMMUS)

Ultrasound is widely used in clinic to obtain anatomical information due to its low cost, high temporal resolution, portability, and safety. However, development of ultrasound for molecular imaging is limited. Novel contrast mechanisms are of interest, including MMUS 40,78-80,104. Magneto-motive ultrasound combines conventional ultrasound techniques with an external magnetic field. Following delivery of MNPs into a target tissue, a strong, focused magnetic field is applied to the tissue sample, causing the MNPs, and thus the bound tissue, to move toward the magnetic source. Ultrasound then detects the displacement of the particle-infused tissue by changes in the speckle pattern. Data can be collected at high frame rates and processed using a speckle tracking algorithm to monitor motion frequency. The difference in frequency between magnetically-labeled tissue and the unlabeled background tissue can distinguish molecular imaging targets. Magnetically-labeled tissue displacement also changes as a function of concentration of the MNPs, which can provide diagnostic information. Magneto-motive ultrasound encompasses the benefits of conventional ultrasound with the added ability to capture molecular-level, functional information in real-time at clinically-relevant penetration depths. Thus far, MMUS has been demonstrated in phantom 40,49,78,79,104, cell 40, and animal studies 80,102.

**Figure 2:**
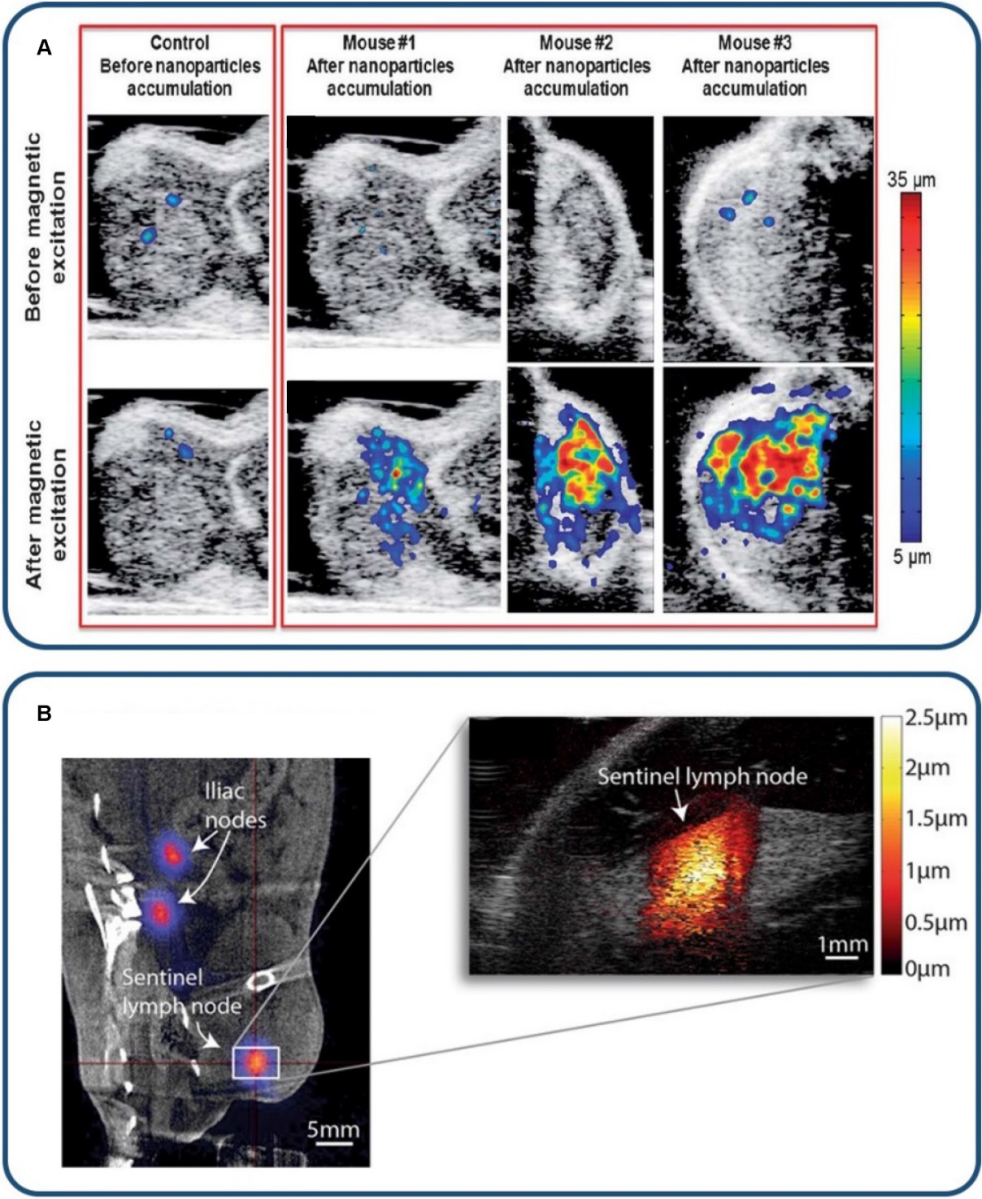
** MMUS/pMMUS for *in vivo* imaging.** (A) *In vivo* US/pMMUS images of mice with A431 tumors. pMMUS signal detected tumors loaded with magnetic nanoparticles. Reproduced with permission from 105. Copyright 2013, Royal Society of Chemistry. (B) *In vivo* PET/CT image (left panel) of ^68^Ga-labelled SPION drainage to the sentinel lymph node. The white box indicates the magnified region in the MMUS image (right panel), where the induced magnetomotive displacement is indicated by the adjacent red color scale. Reproduced with permission from 77. Copyright 2017, Springer Nature.

Pulsed magneto-motive ultrasound (pMMUS) was developed to counteract potential drawbacks of MMUS imaging, specifically thermal deposition 22,33,105,106. Continuous application of a strong magnetic field to image deep tissue targets can cause the magnetically-labeled tissue to generate heat, resulting in thermal damage. In pMMUS, the magnetic field is switched on and off, and the MNPs will realign each time the field is applied. This will cause the particles to pulse and briefly move toward the magnetic source, creating contrast between labeled and unlabeled tissues while minimizing risk of bulk heating. The time-gated pulsatile motion is quantified as the maximum displacement between the first two peaks of the bipolar motion to represent pMMUS signal at each pixel. Figure 2 (top panel) demonstrates the use of pMMUS for tumor imaging *in vivo* 105.

Harmonic MMUS imaging was investigated to identify accumulation of SPIONs in the sentinel lymph node (SLN) *in vivo* (Figure 2 - bottom panel). Evertsson et al. investigated a potential clinical scenario for intra-surgical guidance of metastatic lymph node removal 77. ^68^Ga-labelled MNPs were subcutaneously injected in rats, followed by PET and MMUS imaging at SLNs. Results showed that PET imaging had higher sensitivity and detected lower concentration of contrast agents; however, MMUS performed reasonably well, detecting two-thirds of SLNs. Overall PET and MMUS were found to have distinct advantages in this application. Positron emission tomography is better suited for pre-operative monitoring. As a radiation free, portable imaging modality, MMUS is better suited for real-time intra-operative or bedside imaging with high spatial resolution.

### Magneto-motive OCT (MM-OCT)

**Figure 3:**
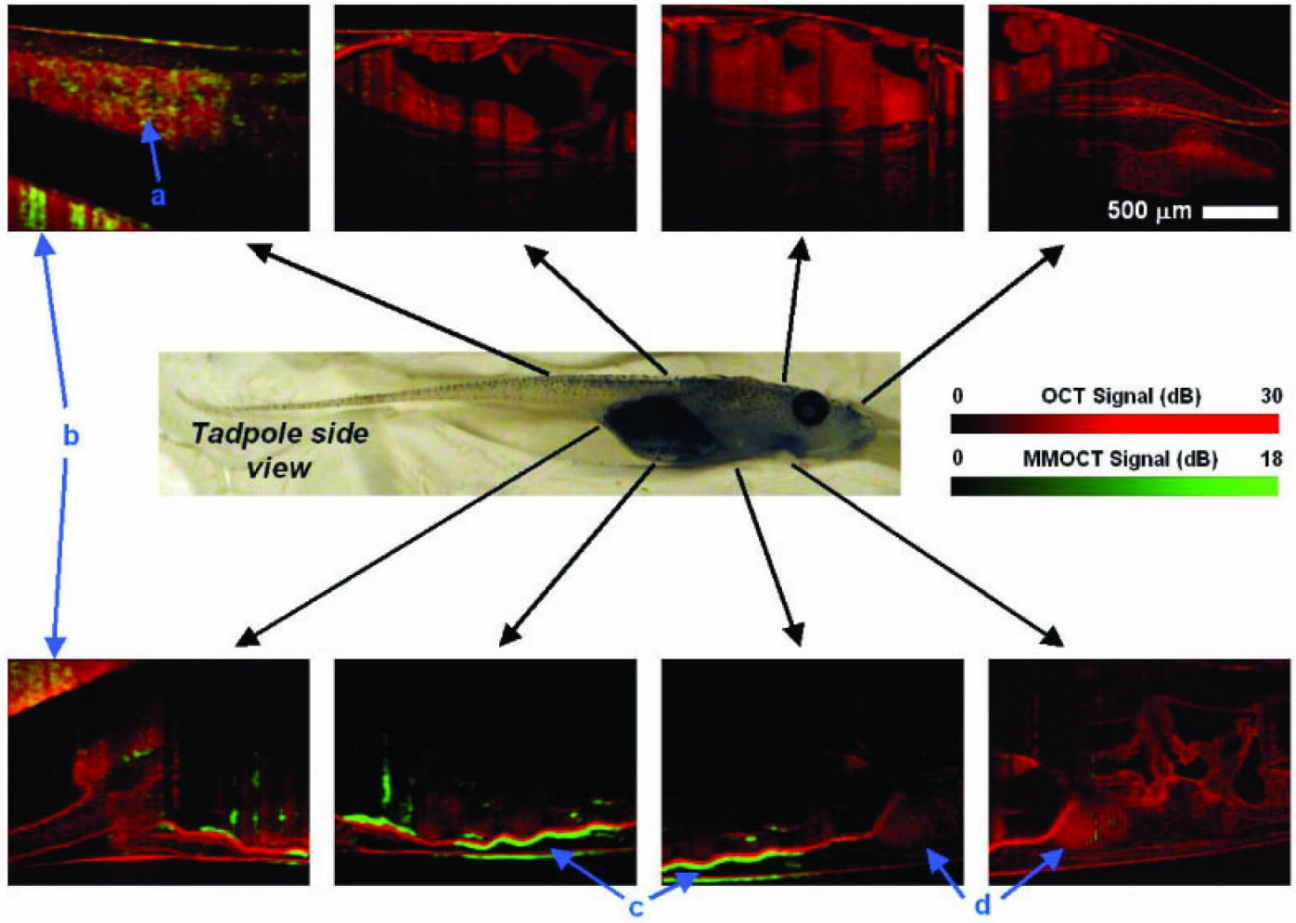
***In vivo* MM-OCT imaging of magnetic nanoparticles in a Xenopus tadpole model fed with magnetic nanoparticles.** Red colormaps represent the structural (no contrast) OCT and the overlaid green signals represent magneto-motive contrast enhanced OCT. Blue arrows with letter identifiers indicate the tail, clay imaging holder, intestines, and heart, respectively. MNPs were primarily observed in the intestines (c). Reproduced with permission from 32. Copyright 2005, Optica Publishing Group.

Optical coherence tomography is widely used for ophthalmic imaging in clinic, particularly for assessing retinal anatomy 107, and is gaining popularity in cardiovascular applications 108-112. Similar to ultrasound, contrast in OCT is generated by backscattered light to create high resolution cross-sectional images of tissue anatomy. However, molecular imaging capabilities of OCT are limited 113,114. Magneto-motive OCT (Figure 3) can detect motion of MNP-labeled targets upon introducing an external magnetic field 32,81-89. OCT uses short wavelengths of light, making MM-OCT favorable for detecting small magnetically-induced displacement. Thus, MM-OCT may allow imaging with higher sensitivity to enhance diagnostic value.

Magnetic microspheres have been used as a dynamic contrast agent for molecular-specific MM-OCT in a variety of applications, including tumor imaging *in vivo* (Figure 3) 32,115,116, platelet imaging 87,88, and stem cell imaging 89. The use of MM-OCT is particularly appealing to allow assessment of tissue microstructure *in vivo*. This type of assessment is typically done through invasive, destructive, histological processing; however, high specificity and background suppression using MM-OCT may provide an excellent alternative. One application of MM-OCT is molecular or cellular characterization in intravascular applications to assess plaque vulnerability 117-119. For example, macrophages or lipid can be labeled with functionalized MNPs to gain further insight on the status of an atherosclerotic lesion using MM-OCT. This was demonstrated *ex vivo* in perfused aortas from an atherosclerotic rabbit model 119. In another application, MM-optical Doppler tomography (MM-ODT) was demonstrated *in vivo* to detect SPIONs implanted in melanoma tissue. The work demonstrates the potential to apply MM-ODT for melanoma detection and the application of Doppler-based methods to detect MNPs with high sensitivity in tissue targets where no physical flow is present 116.

Like all magneto-motive imaging modalities, one drawback of MM-OCT is the need for mechanical resistance of tissue, which can limit use in fluid environments. In the case of cardiovascular applications, movement of blood and the lower restoring force compared to other tissues may limit feasibility of MM-OCT. However, a dual-coil solenoid configuration has been developed to facilitate MM-OCT in samples without elastic restoring force, i.e. liquid samples 83. The ability to measure viscoelastic properties in primarily liquid samples with weak restoring force, such as blood or lymph, extends capabilities of MM-OCT. MM-OCT has also been used to detect blood flow based on temporal analysis of signals from iron-containing hemoglobin 120. Similar approaches may also be applicable to other magneto-motive modalities. A drawback specific to MM-OCT is limited penetration depth of light. In spite of this, MM-OCT has still been demonstrated for *in vivo* applications. Use of longer wavelengths or improved light delivery strategies may increase penetration depth, but other magneto-motive imaging modalities may be better suited to deep applications.

### Magneto-motive laser speckle tracking (MM-LST)

Laser speckle tracking involves the analysis of raw speckle reflectance images collected during the optical excitation of an object 121-126. Various algorithms are used to convert these images into maps of speckle contrast. Advantages of LST compared to other systems include high spatial and temporal resolution, ease of implementation, and relatively low cost. Laser speckle tracking is often used to study blood flow dynamics associated with laser therapy, blood flow dynamics associated with focal cerebral ischemia 124, arterial ventricular flow dynamics 122, and tissue biomechanics with speckle motion tracking 126. Laser speckle tracking can also characterize microvascular parameters such as vessel diameter. One intrinsic limitation of LST for resolving microvascular architecture is that signal depends on the relative motion of red blood cells, which is slow in small vessels. Furthermore, optical scattering during tracking of subsurface blood flow creates additional difficulty for resolving small vessels. Due to the limitation of LST for sensitive detection of regions of slow flow in small vessels, researchers have investigated the use of SPIONs to enhance LST with magneto-motive contrast 94,95. In a phantom study, SPIONs were flowed through a microfluidic chip to assess benchtop feasibility of MM-LST for assessing slow flow environments. Results indicated that MM-LST may have future relevance for detecting tumor vasculature, where blood flow is slow 95.

### Magneto-motive photoacoustic (MPA) imaging

Photoacoustic imaging combines advantages of light and sound by relying on the photoacoustic effect 127-131. Upon irradiation of an optical absorber with a nanosecond pulsed laser, thermoelastic expansion of the surrounding tissue creates an acoustic wave that can be detected by a conventional ultrasound transducer. Unlike ultrasound, absorption-based PA images convey cellular or molecular information with high contrast. However, ultrasound and PA imaging are often combined to convey complementary anatomical and functional information. A variety of optical absorbers can be used as PA contrast agents. The use of exogenous absorbers, such as nanoparticles or dyes, is particularly important for *in vivo* imaging to distinguish background PA signals from endogenous absorbers, including melanin, oxygenated and deoxygenated hemoglobin, and lipid. Multi-wavelength PA imaging followed by spectral analysis can be used to distinguish optical signatures from endogenous and exogenous absorbers. Alternatively, dynamic contrast mechanisms can distinguish absorbers, further suppress background signals, and enhance contrast. One example is MPA imaging. A key aspect of MPA imaging is the use of hybrid nanoparticles that simultaneously possess optical and magnetic properties. Examples include core-shell photomagnetic nanoparticles or liposomes loaded with gold nanorods and iron oxide nanoparticles 34,93. Magneto-motive PA images are produced by combining PA and MMUS imaging 34,36,66,90-93. The most common method to generate MPA images is to use an MMUS map as a mask for PA images to reduce background noise and gain contrast resolution from PA 34,36.

**Figure 4:**
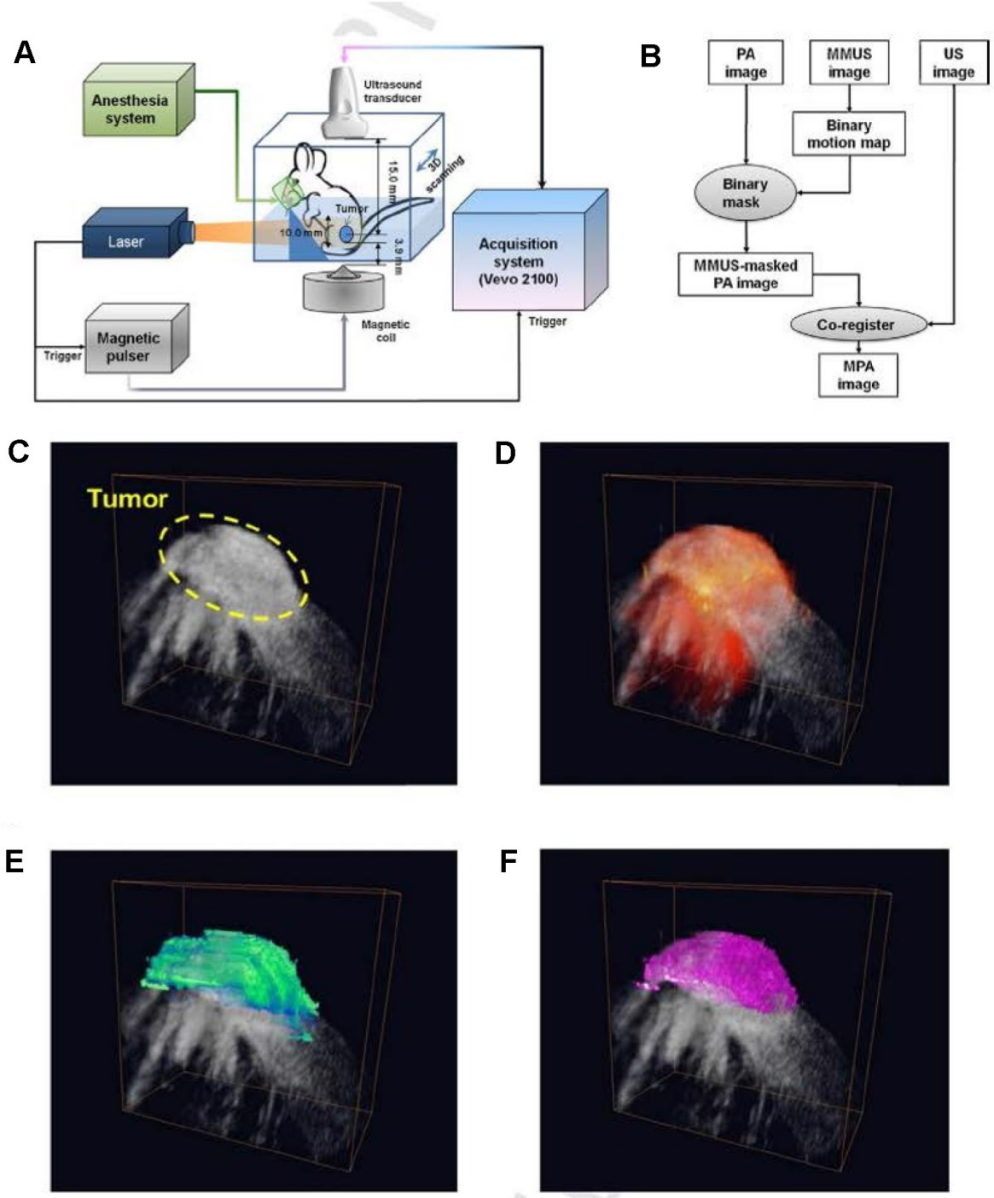
** Magneto-motive PA imaging (MPA) imaging of a tumor *in vivo*.** (A) Block diagram of the *in vivo* MPA imaging system. (B) MPA image formation algorithm. (C) ultrasound (grayscale), (D) PA, (E) MMUS, and (D) MPA images of the tumor loaded with photomagnetic nanoparticles. Reproduced with permission from 36. Copyright 2014, Elsevier GmbH.

In MPA imaging, optical absorption from the optical component of the particle will generate a PA signal of interest. Photoacoustic signals will also be generated from endogenous, background optical absorbers. Ultrasound images are simultaneously acquired to provide anatomical context. Next, an external magnetic pulse creates motion due to the magnetic component of the particle. The magnetic-induced motion changes the speckle pattern of the ultrasound image to produce an MMUS image. The MPA image is ultimately generated by masking the PA image using the MMUS image, which suppresses background PA signals to enhance detection of the photomagnetic nanoparticles (Figure 4) 36.

The benefits of MPA imaging are improved specificity and potential for quantitative imaging. Specificity is improved by utilizing information from two contrast mechanisms, PA and MMUS. The added optical contrast in MPA imaging can also provide quantitative information because nanoparticle concentration directly corresponds to PA signal. Figure 4 depicts the value of combining ultrasound (Figure 4C), PA (Figure 4D), MMUS (Figure 4E), and MPA imaging (Figure 4F) by detecting photomagnetic nanoparticle accumulation in a mouse tumor *in vivo*. Different information is provided by each modality. The MPA image distinguishes the tumor margins, further suppresses background signals, and has enhanced signal from the photomagnetic nanoparticles 36.

Motion artifacts are a common problem for *in vivo* MPA imaging, similar to MMUS. Cycling the magnetic field between the breath and heart rate can minimize motion artifacts, but this is not trivial. A fast repetition rate laser could allow use of PA signal for motion analysis to generate improved MPA images that account for background motion. However, commonly used lasers in PA imaging typically have a low repetition rate. The resulting low temporal resolution limits PA imaging for accurate motion tracking because magneto-motive excitation applies a sharp pulse at moderate to high cycling frequencies.

### Magneto-motive tissue elastography

Magneto-motive imaging techniques can also be employed to gather functional diagnostic information. One example is to evaluate tissue mechanical properties through elastography 132,133. Elastography has become a clinically relevant tool for diagnosing various pathologies that cause changes in tissue elasticity, including liver fibrosis 134,135, breast cancer 136,137, and thyroid cancer 138. Thus, a wide variety of techniques have been developed to assess tissue elasticity, including ultrasound elastography (UE) 133,137-139, magnetic resonance elastography (MRE) 134,135,140, vibro-acoustography 132,133,141,142, and optical coherence elastography (OCE) 143,144.

Elastography methods typically apply an external mechanical stimulus to deform the tissue 145-148. Alternatively, an internal mechanical stimulus can be applied using magneto-motive force, which may allow evaluation of mechanical properties of deeper tissues 103. More specifically, shear wave propagation, i.e. velocity, varies according to elastic properties of tissue, and magnetically-induced tissue motion is a suitable option to generate shear waves 149,150. Pulsed magnetic excitation can generate shear waves for localized tissue elastography. Harmonic magnetic excitation or sweeping excitation frequency can be used to find the natural resonance frequency of a tissue target to reveal mechanical properties 99,151,152. Source frequency sweeping can improve signal-to-noise ratio. However, longer image acquisition time decreases temporal resolution. Another challenge of source frequency sweeping is achieving strong magnetic excitation at higher frequencies due to the frequency-dependent impedance of the excitation coils. The increased impedance at higher frequencies reduces current, and ultimately, the strength of the excitation magnetic field. Alternatively, measuring the response of iron-laden tissue to step-wise magnetic excitation can reveal viscoelastic properties of tissues, where rapid initial displacement of MNPs may be followed by distinct oscillation damping and onset of creep 96,97,100,153. The principles described above have been utilized in magneto-motive optical coherence elastography (MM-OCE) and magneto-motive ultrasound elastography (MM-UE).

**Figure 5:**
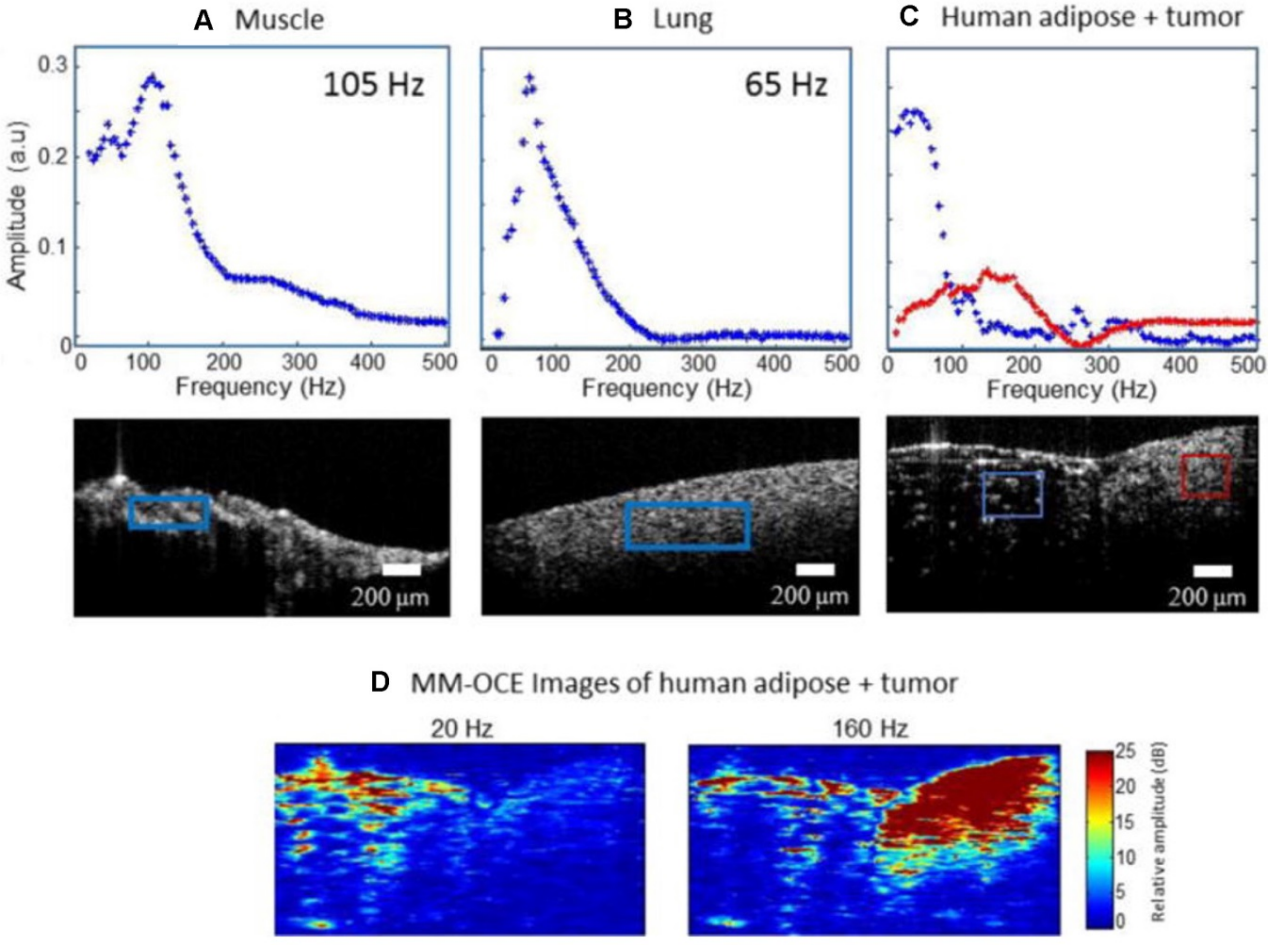
** Magneto-motive optical coherence elastography**. Mechanical resonance frequencies in rat and human tissue samples. (A) Rat muscle and (B) rat lung tissue. (C) Human adipose tissue and tumor. The red and blue boxes indicate the spatial regions from which the displacement amplitudes shown in the plots were calculated. (D) MM-OCE images of human adipose tissue and tumor. At low magnetomotive frequency (20 Hz shown) the adipose is highlighted. At higher frequencies (160 Hz shown) the stiffer tumor region gives a higher magnetomotive signal. Reproduced with permission from 99. Copyright 2015, IOP Publishing.

MM-OCE is appealing due to its high resolution to measure tissue biomechanical properties at the microscale 88,96-99. The ability to apply an internal magnetic stimulus is particularly beneficial in MM-OCE compared to standard OCE, which suffers from shallow imaging depth. Furthermore, the use of an internally generated magnetic force can provide localized mechanical perturbations to better assess the tissue microenvironment. Thus far, MM-OCE has primarily been assessed in phantoms, *in vitro*, or *ex vivo,* for example in models of blood clots or cystic fibrosis assessment.^88^,^98^ Figure 5 shows feasibility of MM-OCE using a spectral domain approach to assess tissue phantoms with heterogenous stiffness. The soft region of the phantom showed higher magneto-motive response at low mechanical excitation frequencies, whereas the stiff region showed higher magneto-motive response at high mechanical excitation frequencies 99.

Recent research shows the first successful *in vivo* demonstrations of MM-OCE 149,151. Previously, one major constraint of MM-OCE for *in vivo* applications was long image acquisition, which was caused by long inter-frame wait time to prevent coil overheating from repeated magnetic excitation. By instead using a broadband magnetic force in a chirped waveform, mouse skin was imaged *in vivo* and results demonstrated an increased speed of at least 414x for acquisition and 131x for post-processing compared to previous methods 149. In another study, MM-OCE was employed to assess heat-induced stiffness changes following magnetic hyperthermia treatment in a melanoma mouse model, showing theranostic potential of MNPs combined with MM-OCE 151.

Magneto-motive ultrasound elastography can also be used to analyze tissue elasticity. Similarly, a number of studies have been conducted in tissue-mimicking phantoms of varying stiffness containing MNPs 100-103. In one study using pMMUS, increasing sample stiffness, and therefore the shear modulus, resulted in lower displacement of MNPs 100. To increase pMMUS contrast enhancement and sensitivity to variations in tissue stiffness, a recent study proposed using microbubbles labeled with SPIONs to generate larger displacements by coupling magneto-induced motion and bubble oscillations 154. Another approach to assess viscoelastic properties of soft tissues is to induce shear waves via MNP motion and track wave propagation, termed shear wave dispersion magneto-motive ultrasound (SDMMUS) 101. Differences in shear wave velocity and attenuation correlate with differences in elasticity and viscosity of the medium. In general, SDMMUS systems are more expensive due to more advanced hardware requirements to track shear waves. As a more cost-effective alternative, researchers are developing platforms to integrate MMUS and SDMMUS imaging with existing clinical systems 49,102.

## Future outlook of magneto-motive imaging modalities

Given the biosafety of MNPs and the established use of magnetic excitation in clinic, magneto-motive imaging modalities have great potential to become clinical tools for contrast-enhanced molecular imaging and therapy monitoring. Design of functionalized MNPs with specific targeting moieties has been widely studied for decades, which sets the stage for development of magneto-motive imaging modalities as highly sensitive and specific molecular imaging techniques. In addition, therapeutic applications of MNPs, such as drug delivery and magnetic hyperthermia, expand the utility of magneto-motive imaging as a theranostic tool to monitor treatment progression and outcomes. Advances in electronic hardware, data acquisition, and signal processing methods for displacement measurements, can further promote magneto-motive imaging in the future.

Despite the potential of magneto-motive imaging modalities, clinical translation remains limited. Translation may be expedited by first using magnetic contrast agents that are already FDA-approved or integrating magneto-motive imaging techniques with familiar, clinically established modalities. One challenge in extending magneto-motive imaging beyond the pre-clinical stage is insufficient contrast at greater penetration depths. This magnifies the difficulty of distinguishing magnetically-induced motion from physiological tissue motion, i.e. cardiac or respiratory. Therefore, any effort to enhance magneto-motive contrast is critical. The remaining sections discuss the future outlook of magneto-motive imaging, as well as key opportunities for further development towards theranostic applications and clinical translation.

### Contrast enhancement through magnetic contrast agents

There are two main strategies to develop MNPs with enhanced contrast for magneto-motive imaging. The first strategy is to develop MNPs with greater magnetic susceptibility (*χ_np_*). Metal-doped iron oxide nanoparticles, such as zinc, cobalt, or nickel, have been introduced to enhance magneto-motive force, and therefore, magneto-motive contrast 105,155,156. The second strategy is to develop large MNPs (*V_np_*) with a greater fraction of magnetic material (*f_m_*). However, simply increasing the core size of single MNPs is not feasible given the physical size constraints to maintain superparamagnetic properties. Therefore, researchers developed magnetic nanoclusters 105. The nanoclusters are composed of individual SPIONs that are bound to each other through encapsulation in a large carrier particle or through clustering via a degradable polymer 105. While these constructs maintain superparamagnetic properties, their effective volume is increased through clustering to improve magneto-motive contrast.

### Enhanced and focused magnetic excitation

Spatial degradation of the magnetic field over the imaging distance is another challenge for clinical implementation of magneto-motive imaging systems. A spatial gradient is a key factor in generating magneto-motive force. However, B(z) and its gradient degrade at greater distances from the magnetic excitation source, and, as a result, there is insufficient magneto-motive force to move MNP-labeled tissue. This limits the accessible penetration depth of magneto-motive imaging modalities, especially when using a single excitation source. One potential solution is to use a multiple-coil system to focus the magnetic field deep inside the tissue, which is used to localize magnetic excitation to the brain with transcranial magnetic stimulation systems 157,158. Similar strategies can be used in magneto-motive imaging to overcome field degradation and to increase magneto-motive imaging depth 38.

Safety needs to be considered when developing strategies to focus or enhance the magnetic field. Magnetic excitation parameters in MRI or MPI can be used to determine safe constraints for magneto-motive imaging. The safety of MRI scanners for human scans can be divided into the safety of the static or gradient fields. In terms of the static field, 3T scanners are currently used in clinical practice. Magnetic field strength (peak magnetic flux densities) of magneto-motive imaging modalities is significantly smaller than 3T, indicating no safety concerns related to the maximum flux density 38,74,116,159,160. In terms of gradient fields, MRI machines utilize magnetic field gradients with amplitudes on the order of 100 mT/m, with slew rates up to 200 mT/m/ms 160-162. The magnetic field gradients used in magneto-motive imaging are often below the aforementioned limits. However, systematic study is required, especially for fast magneto-motive excitation, where peripheral or cardiac nerve stimulation could occur 163. The magnetic field strength and gradient of magneto-motive imaging modalities is also typically smaller than MPI systems (for example, 6.1 T/m used in the MOMENTUM system; Magnetic Insight, Alameda, California). As with any imaging modality, magneto-motive imaging will not be appropriate for all patients or all procedures due to potential interference with cardiac function or generation of heat, for example at locations of catheters or guide-wires 164. However, based on similarities to MRI and MPI, when magnetic fields are generated following appropriate constraints, magneto-motive imaging modalities are safe.

### Accurate detection of magneto-motive contrast in presence of biological motion

Physiological motion of subjects due to cardiac and respiratory functions degrades the quality of magneto-motive images 38. Motion artifacts are an issue for most existing imaging modalities. However, because contrast in magneto-motive imaging is directly derived from tissue motion, non-magneto-motive motion is a significant obstacle in distinguishing real magneto-motive signal. Methods to compensate for tissue motion and reduce motion artifacts have been extensively investigated in other applications. Research has demonstrated the efficacy of motion gating techniques to reduce artifacts 165-167. These techniques utilize electrocardiogram and respiratory monitoring systems to define an ideal time window for image acquisition, when the subject has the least motion.

In harmonic magneto-motive imaging (i.e. using continuous cyclic sinusoidal magnetic excitation), the fundamental frequency of magnetically induced motion is twice the excitation frequency of the externally applied magnetic field 40,104. Therefore, the magneto-motive signal can be extracted by bandpass filtering. In the case of pulsed or non-sinusoidal excitation, magnetic excitation pulses are short with sharp transitions. As a result, magnetically induced motion has a sharp temporal response, providing another opportunity for motion-gating strategies. Sharp, fast magneto-motive motion can be separated from gradual, slow tissue motion by analyzing frequency content. Developing more accurate and sensitive motion detection algorithms, such as frequency or phase locking and blind-source separation, can enhance the sensitivity of magneto-motive imaging and reduce background noise 79,168.

### Clinical translation as a complement to existing modalities

Integration with existing clinical systems may expedite clinical translation of magneto-motive imaging techniques by providing complementary information while minimizing changes to current imaging protocols. Thus, magneto-motive imaging can be more seamlessly added to the current clinical landscape. One example was previously discussed, where clinically-available ultrasound systems were adapted for MMUS and SDMMUS to provide anatomical context along with complementary molecular information and tissue characterization 102. Integration of OCT or ultrasound with their magneto-motive counterparts generally has two requirements: 1) hardware must support the image acquisition speed necessary for magneto-motive contrast; 2) an external magnetic force must be integrated with the system. Magneto-motive PA imaging may be viewed as a more complex multi-modal imaging approach, requiring integration of ultrasound, PA, and MMUS imaging. However, the appeal of MPA is the additional sensitivity and specificity of using a hybrid technique with optical and magnetic contrast. In the future, multimodal MPA approaches can be further developed into theranostic platforms. One example includes monitoring magnetically-induced hyperthermia for cancer treatment 169.

### Theranostic applications of magneto-motive imaging

Magnetic motion of MNPs can simultaneously be leveraged for treatment and imaging to create many theranostic opportunities, including image-guided magnetic drug delivery or image-guided cancer therapies. Magnetic nanoparticles have been widely used as carriers for targeted drug delivery. Magneto-motive imaging modalities are perfect tools to verify the accumulation of NP-loaded drugs at the target 170-172. Furthermore, magneto-motive imaging modalities have potential to monitor intracellular accumulation of NP-loaded drugs, which is central to effective drug delivery 90.

In cancer therapies, MNPs can serve multiple functions. First, based on their small size, MNPs will passively accumulate at tumor sites due to the enhanced permeability and retention (EPR) effect. The EPR effect naturally accumulates nano-sized particles at the tumor via leaky vasculature, while preventing escape due to lack of lymphatic drainage 173. However, the EPR effect results in very heterogeneous distribution of nanoparticles, whereas homogenous distribution is desired to more effectively treat the entire tumor 46. Instead, MNPs can be actively targeted to the tumor via an external magnetic field. Magneto-motive imaging can then verify and monitor MNP accumulation for downstream treatment, such as hyperthermia.

The goal of hyperthermia for cancer therapy is to cause a temperature increase above 41˚C at the tumor to impact cells at the DNA, protein, or enzymatic level to induce apoptosis 173. For magnetic hyperthermia, heat is generated by applying an alternating magnetic field to MNPs. The MNPs absorb this energy and convert it to heat upon relaxation of the magnetic moments 174,175. Details on this mechanism are further described elsewhere 175. Magnetic hyperthermia treatments and magneto-motive imaging can be integrated to monitor hyperthermia dose and assess changes in biomechanical properties of tissues 151,176. During hyperthermia treatments, the motion of MNPs will change in response to local variations in tissue biomechanical properties due to heating. Therefore, magneto-motive signal can represent tissue response to therapy.

As a multimodal approach, MPA imaging can facilitate tumor detection with high sensitivity and high specificity, while simultaneously leveraging light and sound for therapy. After verifying accumulation of photomagnetic nanoparticles at the tumor via MPA, hyperthermic cell death can be induced with light, magnets, or a combination of both. Thermal ultrasound or thermal PA imaging can provide temperature maps to assure the tumor reaches the desired treatment temperature 176,177. Following initial treatment, MM-UE can monitor cell death or tissue ablation based on changes in elastic properties. Multi-wavelength PA imaging and spectral analysis could provide additional diagnostic information on treatment status by assessing tissue oxygen saturation 178,179. A similar theranostic approach was demonstrated with MM-OCE. The magnetic thermotherapy dose and the resulting tissue ablation were monitored based on changes in elasticity 180,181.

Outside of applications in guiding drug delivery or cancer therapy, magneto-motive displacement was applied at the tympanic membrane to enhance sound perception 182. Application of iron oxide nanoparticles at the rat tympanic membrane (TM), *ex vivo*, caused increased vibration of the TM upon exposure to an alternating magnetic field. Manipulation of the amplitude and frequency of the vibrations was monitored using OCT. This platform may be used to enhance sound perception, while magneto-motive imaging can monitor/verify hearing recovery to adjust treatment.

**Figure 6:**
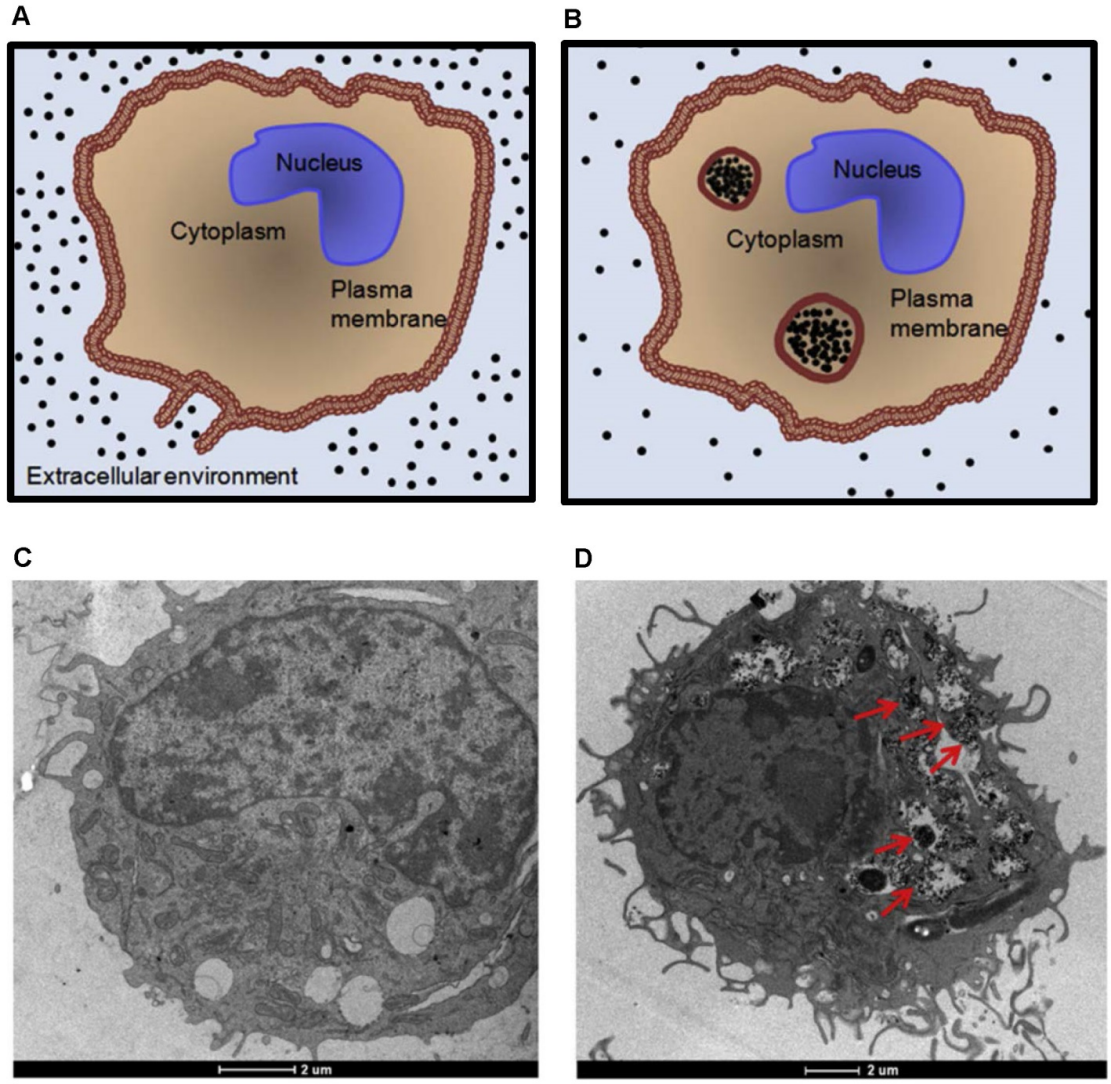
** Accumulation of magnetic nanoparticles within cells.** (A) and (B) Diagrams depicting intracellular uptake of magnetic nanoparticles by phagocytic cells. The size of the endosomes can be as large as 5 µm. TEM images of unlabeled (C) and SPION-labeled macrophages (D). Red arrows indicate the endocytosed aggregates of SPIONs within endosomes. Reproduced with permission from 106. Copyright 2011, IOP Publishing.

Lastly, studies indicate significant magneto-motive signal enhancement due to accumulation of MNPs within cells 106. The diagram and transmission electron microscopy images in Figure 6 depict accumulation of MNPs within cells. Due to their small size, when individual MNPs are not accumulated within cells or do not interact with their surroundings, particles may move freely upon external magnetic excitation. In other words, unconfined MNPs are simply pulled through tissue to a new location, rather than experiencing the necessary viscoelastic resistance to generate a magneto-motive signal. However, when MNPs are accumulated inside endosomes, cells, or interact with their surroundings, movement is confined. Therefore, magneto-motive signal will increase and change due to enhanced engagement of MNPs with their environment. This effect is important in understanding, manipulating, and sensing different interactions between MNPs and organelles, cells, or tissues towards development of magneto-motive theranostic platforms for biomedical applications.

## Conclusions

Magneto-motive imaging techniques have received great interest due to detection with high contrast, sensitivity, and specificity. By utilizing magnetically-induced motion as a contrast mechanism, the use of traditionally non-magnetic modalities can be expanded. This review described magnetic nanoparticle design, which is essential to effectively generating magneto-motive contrast, and provided an overview of current magneto-motive imaging techniques, including MMUS, MM-OCT, MPA, and MM-LST. Beyond diagnostic imaging, functional imaging using MM-UE and MM-OCE was described to assess tissue elastography. At this point, magneto-motive imaging has not yet reached clinical translation, but a variety of opportunities exist to motivate future development and translation of these exciting imaging tools.

## Abbreviations

CT: computed tomography
EPR: enhanced permeability and retention
LST: laser speckle tracking
MM: magneto-motive
MM-OCE: magneto-motive optical coherence elastography
MM-OCT: magneto-motive optical coherence tomography
MM-UE: magneto-motive ultrasound elastography
MMLST: magneto-motive laser speckle tracking
MMUS: magneto-motive ultrasound
MPA: magneto-photoacoustic
MRE: magnetic resonance elastography
MRI: magnetic resonance imaging
MNP: magnetic nanoparticle
OCT: optical coherence tomography
OCE: optical coherence elastography
PA: photoacoustic
PET: positron emission tomography
pMMUS: pulsed magneto-motive ultrasound
SDMMUS: shear wave dispersion magneto-motive ultrasound
SLN: sentinel lymph node
SPION: superparamagnetic iron oxide nanoparticle
TM: tympanic membrane
UE: ultrasound elastography
US: ultrasound
